# Folic acid conjugation improves the bioavailability and chemosensitizing efficacy of curcumin-encapsulated PLGA-PEG nanoparticles towards paclitaxel chemotherapy

**DOI:** 10.18632/oncotarget.22376

**Published:** 2017-11-10

**Authors:** Arun Kumar T. Thulasidasan, Archana P. Retnakumari, Mohan Shankar, Vinod Vijayakurup, Shabna Anwar, Sanu Thankachan, Kavya S. Pillai, Jisha J. Pillai, C. Devika Nandan, Vijai V. Alex, Teena Jacob Chirayil, Sankar Sundaram, Gopalakrishnapillai Sankaramangalam Vinod Kumar, Ruby John Anto

**Affiliations:** ^1^ Division of Cancer Research, Rajiv Gandhi Centre for Biotechnology, Thiruvananthapuram, Kerala, India; ^2^ Division of Chemical Biology-Nano Drug Delivery Systems, Rajiv Gandhi Centre for Biotechnology, Thiruvananthapuram, Kerala, India; ^3^ Research Scholar, University of Kerala, Thiruvananthapuram, Kerala, India; ^4^ Research Scholar, Manipal University, Manipal, Karnataka, India; ^5^ Department of Pathology, Government Medical College, Kottayam, Kerala, India

**Keywords:** curcumin, folic acid conjugation, PLGA nanoparticles, bioavailability, chemosensitization

## Abstract

Nanoencapsulation has emerged as a novel strategy to enhance the pharmacokinetic and therapeutic potential of conventional drugs. Recent studies from our lab have established the efficacy of curcumin in sensitizing cervical cancer cells and breast cancer cells towards paclitaxel and 5-FU chemotherapy respectively. Factors that hinder the clinical use of curcumin as a sensitizer or therapeutic agent include its poor bioavailability and retention time. Earlier reports of improvement in bioavailability and retention of drugs upon nanoencapsulation have motivated us in developing various nanoformulations of curcumin, which were found to exhibit significant enhancement in bioavailability and retention time as assessed by our previous *in vitro* studies. Among the various formulations tested, curcumin-entrapped in PLGA-PEG nanoparticles conjugated to folic acid (PPF-curcumin) displayed maximum cell death. In the present study, we have demonstrated the efficacy of this formulation in augmenting the bioavailability and retention time of curcumin, *in vivo*, in *Swiss albino* mice. Further, the acute and chronic toxicity studies proved that the formulation is pharmacologically safe. We have also evaluated its potential in chemosensitizing cervical cancer cells to paclitaxel and have verified the results using cervical cancer xenograft model in NOD-SCID mice. Folic acid conjugation significantly enhanced the efficacy of curcumin in down-regulating various survival signals induced by paclitaxel in cervical cancer cells and have considerably improved its potential in inhibiting the tumor growth of cervical cancer xenografts. The non-toxic nature coupled with improved chemosensitization potential makes PPF-curcumin a promising candidate formulation for clinical trials.

## INTRODUCTION

Paclitaxel is one of the most potent naturally derived chemotherapeutic agents discovered till date [1]. It is an anti-mitotic agent that promotes apoptosis in cancer cells by stabilizing microtubules, and has been widely used for treating several cancers [2, 3]. However, its efficacy as an anticancer agent keeps declining as cells become resistant to the normally administered dose, thus creating a need for exposure to higher doses of the drug leading to more deleterious side effects [4]. Pre-clinical and clinical studies have identified multitude of resistance mechanisms such as, altered metabolism of the drug [5], over-expression of the drug transporter p-glycoprotein [6], mutations in the target molecule β-tubulin [7], alterations in the apoptotic signaling mechanisms [5, 7] etc. Owing to the hydrophobic nature of paclitaxel, it is administered in Cremophor EL solvent which has also been reported to induce toxicity. Although, Nab paclitaxel (paclitaxel bound to albumin nanoparticles) could overcome most of the limitations of conventional paclitaxel [8], it is highly expensive and cannot be afforded by common people. Hence, a more feasible and cost-effective option would be to sensitize the cancer cells towards paclitaxel chemotherapy using nontoxic chemosensitizers which when used in combination, bring down the effective dose of paclitaxel to be used. Previous investigations from our lab have illustrated the potential of curcumin, a polyphenol derived from *Curcuma longa*, in chemosensitizing cervical cancer cells to paclitaxel [9–11] and breast cancer cells to 5-flurouracil [12]. Curcumin has been found to be effective in suppressing paclitaxel-induced NF-κB pathway in breast cancer cells and lung cancer cells [13]. However, the major drawbacks in using curcumin as a chemosensitizer *in vivo* were its poor aqueous solubility leading to its fast clearance and poor bio-availability at the target site [14]. Encapsulation of curcumin in nanoparticles has been proved as a feasible strategy to improve the circulation and absorption of highly hydrophobic drugs [15]. Co-administration of paclitaxel and curcumin as nanoemulsions has been shown to overcome multidrug resistance in tumor cells by Ganta S *et al.* [16]. Our *in vitro* studies have successfully demonstrated that, encapsulation of curcumin in PLGA nanoparticles conjugated with folic acid could increase the therapeutic potential of curcumin [17, 18]. In the current study, we have carried out extensive *in vitro* and *in vivo* studies to evaluate the chemosensitizing efficacy of PPF-curcumin towards paclitaxel chemotherapy. We could successfully demonstrate that the encapsulation of curcumin in PLGA-PEG nanoparticles and further conjugation with folic acid enhanced the bioavailability and tissue retention of curcumin *in vivo* compared to liposomal curcumin. We have reported earlier the *in vivo* synergistic efficacy of paclitaxel and curcumin in NOD-SCID mice [11], wherein the route of administration for toxicity and tumor reduction studies were intraperitoneal. Since the present study aimed to evaluate whether folic acid conjugation can improve the tissue retention and bioavailability of curcumin encapsulated PLGA-PEG nanoparticles than liposomal curcumin (as used in the previous study), the same route of administration was used for both tumor reduction and safety studies. Our *in vivo* studies could successfully validate the synergistic efficacy of PPF-curcumin in paclitaxel chemotherapy and the results indicated that PPF-curcumin exhibited a superior efficacy when compared with that of liposome curcumin. Molecular level analyses have shown that PPF-curcumin is much superior in down-regulating paclitaxel-induced up-regulation of survival, proliferative and pro-metastatic signals. We strongly believe that the current study, illustrating the efficacy of PPF-curcumin might be a therapeutically efficient strategy for sensitizing cancer cells towards paclitaxel, which could further enhance the therapeutic outcome of paclitaxel chemotherapy.

## RESULTS

### Encapsulation of curcumin in folic acid conjugated PLGA-PEG nanoparticles significantly improves its efficacy in chemosensitizing HeLa cells

Our earlier studies have already established that curcumin could be used as an effective chemosensitizer in paclitaxel chemotherapy [9–11]. Curcumin encapsulated in nanoparticles prepared from PLGA-PEG block copolymer and conjugated to the tumor-targeting ligand folic acid showed significant chemosensitization potential towards paclitaxel compared to free curcumin [19]. These nanoparticles abbreviated as PPF-curcumin which showed a typical size of 100–200 nm in TEM (Supplementary Figure 1) exhibited a sustained release of curcumin *in vitro*. We have also observed that PPF-curcumin exhibits enhanced therapeutic efficacy *in vitro*, compared to either PLGA-curcumin or free curcumin [19], probably due to uptake of the cucrumin via folate receptors (FOLR1) reported to be over-expressed in almost all cancer types [20].

**Figure 1:**
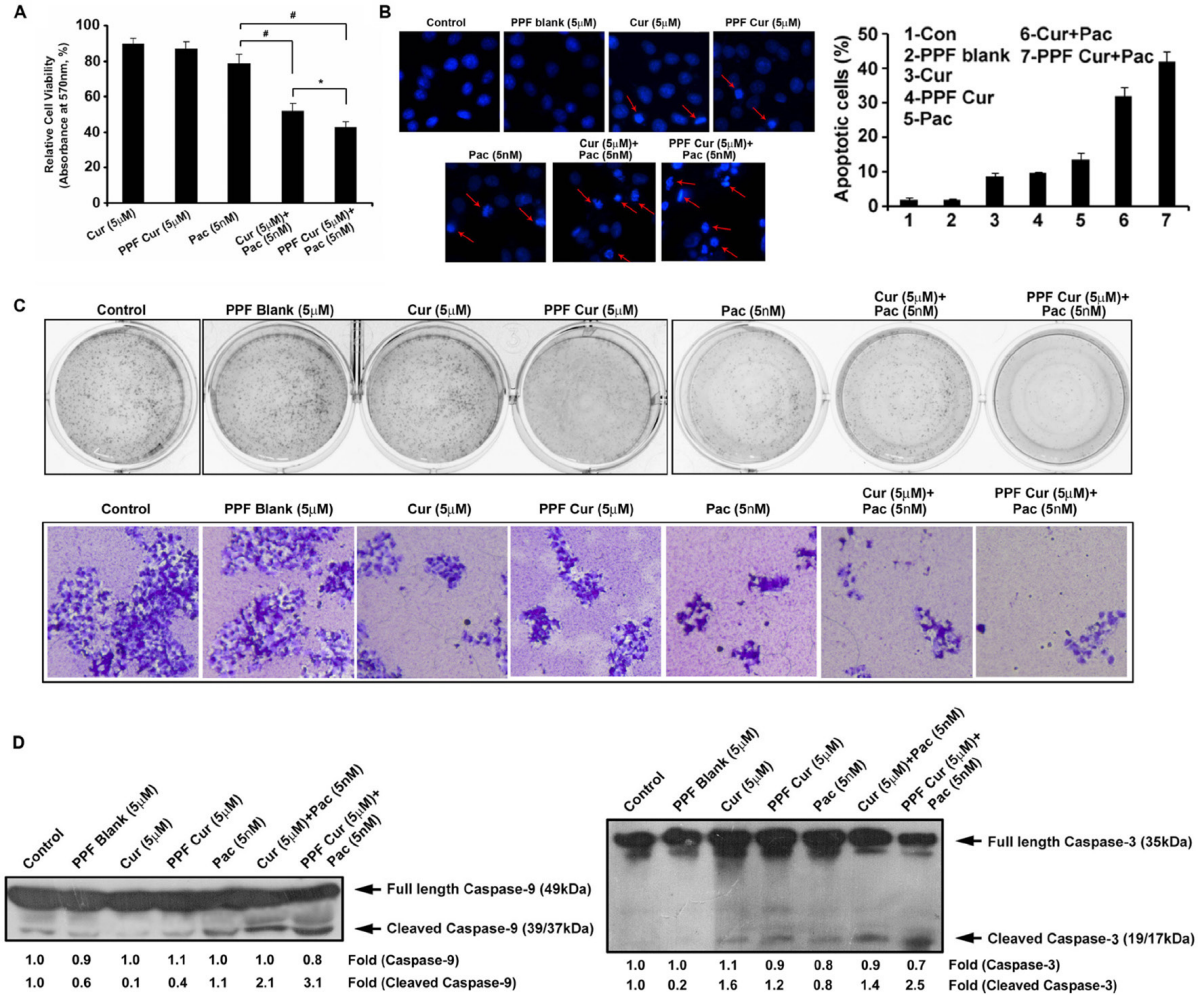
PPF-curcumin induces significant chemosensitization towards paclitaxel (**A**) PPF-curcumin is more efficient in augmenting paclitaxel-induced cytotoxicity in HeLa cells, compared to free curcumin. HeLa cells were treated with free curcumin/PPF-curcumin either alone or in combination with paclitaxel for 72 h after pre-treating with curcumin/PPF-curcumin and cell viability assay was performed using MTT. (**B**) Paclitaxel-induced chromatin condensation is enhanced more efficiently by PPF-curcumin compared to free curcumin. HeLa cells were treated with free curcumin/PPF curcumin either alone or in combination with paclitaxel for 24 h after pre-treating with curcumin/PPF-curcumin. Later, cells were stained with DAPI and images were captured using a fluorescent microscope. The number of condensed nuclei is counted and represented as a graph. (**C**) PPF-curcumin inhibits clonogenicity of HeLa cells. Cells were treated with different formulations of curcumin (5 μM) either alone or in combination with paclitaxel for 72 h and clonogenic assay was conducted. The lower panel shows the images of individual wells captured at 10X magnification. (**D**) Paclitaxel-induced caspase cleavage is enhanced by PPF-curcumin more efficiently than free curcumin. HeLa cells were treated with free curcumin or PPF-curcumin either alone or in combination with paclitaxel for 24h after pre-treating with curcumin/PPF-curcumin, subjected to Western blotting followed by detection by ECL.

In the current study, we compared the synergistic cytotoxicity of free curcumin and PPF-curcumin in combination with paclitaxel, using MTT assay. Figure 1A showed that PPF-curcumin exerts enhanced chemosensitization potential towards paclitaxel-induced cytotoxicity compared to free curcumin. The combination index indicating synergistic cytotoxic effect of free curcumin and PPF-curcumin in combination with paclitaxel was determined using Compusyn software. The results are incorporated in Supplementary Table 1. The combination of 5 nM paclitaxel and 5 μM curcumin induced a synergistic cytotoxicity with a combination index CI of 0.685, whereas the combination of 5 nM paclitaxel and 5 μM PPF-curcumin induced a synergistic cytotoxicity with a combination index CI of 0.315. The chromatin condensation assay showed in Figure 1B indicates significant enhancement in apoptosis as indicated by the extensive condensation of chromatin in PPF-curcumin+paclitaxel treated cells compared to that of free curcumin+paclitaxel. The number of condensed nuclei is counted and represented as a graph. The combination of PPF-curcumin and paclitaxel also caused drastic inhibition in the clonogenic potential of HeLa cells compared to free curcumin as shown in Figure 1C. For improved clarity of the clones, 10x images of the individual wells are shown in the lower panel of Figure 1C. Even though both free curcumin and PPF-curcumin augmented paclitaxel-induced cleavage of caspase-9 and caspase-3, the effect induced by PPF-curcumin was superior to that of free curcumin as indicated by Figure 1D. To make sure that the combination of PPF-curcumin and paclitaxel is not inducing synergistic cytotoxicity towards normal cells, we evaluated their cytotoxicity in the non-tumorigenic immortalized cell line, HaCaT, which has successfully been used earlier as a suitable control by other groups [21]. In contrast to the results obtained in HeLa, neither free curcumin nor PPF-curcumin induced synergistic cytotoxicity in HaCaT cells in combination with paclitaxel (Supplementary Figure 2), corroborating that curcumin or PPF-curcumin does not sensitize normal cells towards paclitaxel, attesting our previous studies using normal cervical cells [9, 11].

### PPF-curcumin is more efficient in down-regulating paclitaxel-induced activation of NF-κB, Akt and MAPK pathways compared to free curcumin

**Figure 2:**
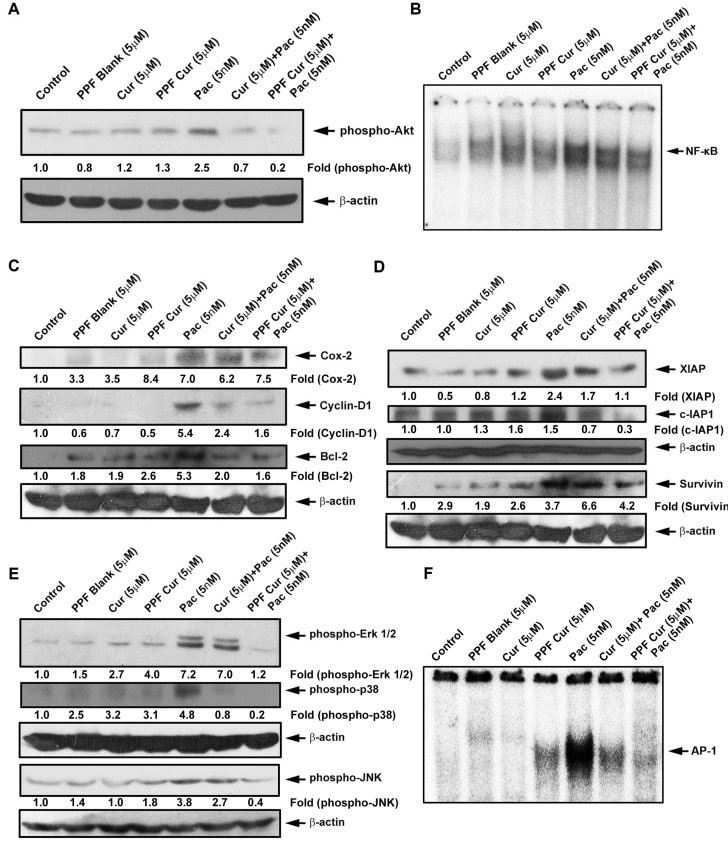
Paclitaxel-induced activation of survival signals is down-regulated more efficiently by PPF-curcumin compared to free curcumin (**A**) Paclitaxel-induced phosphorylation of Akt is more efficiently down-regulated by PPF-curcumin than free curcumin. HeLa cells were treated with free curcumin or PPF curcumin either alone or in combination with paclitaxel for 1 h after pre-treating with curcumin/PPF-curcumin and Western blotting and ECL were performed. (**B**) PPF-curcumin induced significant inhibition of NF-κB activation. HeLa cells were treated with free curcumin or PPF-curcumin for 2 h and/or paclitaxel for 30 min after pre-treating with curcumin/PPF-curcumin, after which nuclear extracts were prepared and EMSA was performed. (**C**) Paclitaxel-induced up-regulation of Cox-2, Cyclin-D1 and Bcl-2 are down-regulated more efficiently by PPF-curcumin than free curcumin. HeLa cells were treated with free curcumin or PPF-curcumin either alone or in combination with paclitaxel for 24 h, after pre-treating with curcumin/PPF-curcumin and Western blotting and ECL were performed. (**D**) Paclitaxel-induced up-regulation of IAPs is more proficiently down-regulated by PPF-curcumin than free curcumin. HeLa cells were treated with free curcumin or PPF-curcumin either alone or in combination with paclitaxel for 24 h, after pre-treating with curcumin/PPF-curcumin and Western blotting and ECL were performed. (**E**) Paclitaxel-induced phosphorylation of Erk 1/2, p38 and JNK are more effectively down-regulated by PPF-curcumin compared to free curcumin. HeLa cells were treated with free curcumin or PPF-curcumin and/or paclitaxel for 15 min, after pre-treating with curcumin/PPF-curcumin and subjected to Western blotting followed by detection by ECL. All the blots were quantified and band density of individual bands are indicated in the blots. (**F**) PPF-curcumin induced significant inhibition of AP-1 activation. HeLa cells were treated with free curcumin or PPF-curcumin for 2 h and/or paclitaxel for 30 min after pre-treating with curcumin, after which nuclear extracts were prepared and EMSA was performed.

The activation of NF-κB, Akt and Mitogen Activated Protein Kinases (MAPK) are common events responsible for the survival, proliferation and drug resistance of tumor cells [22–25]. Abrogation of these pathways using small interfering RNA (siRNA) or inhibitors have been shown to cause reduction in proliferation of cancer cells as well as tumor reduction *in vivo* either alone or in combination with other chemotherapeutic drugs [26–29]. Prolonged exposure of chemotherapeutic drugs including paclitaxel have been shown to activate these survival signals, which in turn make the cancer cells chemo-resistant, necessitating higher doses of the drugs to elicit a desired therapeutic effect [30]. Previous *in vitro* and *in vivo* studies from our group have demonstrated the efficacy of curcumin in successfully bringing down paclitaxel-induced activation of survival pathways in cervical cancer [11]. We questioned whether encapsulation of curcumin in PPF nanoparticles can enhance its ability in down-regulating paclitaxel-induced survival signals. Figure 2A clearly indicates that, PPF-curcumin is much more efficient in down-regulating paclitaxel-induced phosphorylation of Akt compared to free curcumin. Evaluation of the DNA binding of NF-κB by electrophoretic mobility shift assay (EMSA) as shown in Figure 2B also demonstrated that PPF-curcumin is more successful than free curcumin. Paclitaxel-induced NF-κB activation leads to its nuclear translocation ensuing induction of target genes such as cyclin D1, Bcl-2 and Cox-2, all of which can in turn contribute to chemoresistance. In concordance with the EMSA results, PPF-curcumin displayed better efficacy in down-regulating paclitaxel-induced up-regulation of NF-κB target genes such as Cyclin D1, Cox-2, Bcl-2, XIAP, c-IAP and survivin than free curcumin as shown in Figure 2C and 2D. Our earlier studies have revealed the regulatory role of curcumin in modulating paclitaxel-induced activation of MAPK pathway [10, 11]. Figure 2E and 2F clearly indicate that, PPF encapsulation drastically enhances the potential of curcumin in regulating the MAPKs, ERK1/2, JNK and p-38 and their downstream transcription factor, AP-1. Taken together the *in vitro* results clearly demonstrate that, PPF encapsulation significantly enhances the chemosensitizing efficacy of curcumin towards paclitaxel in HeLa cells.

### PPF-curcumin is pharmacologically safe in healthy *Swiss albino* mice

**Figure 3:**
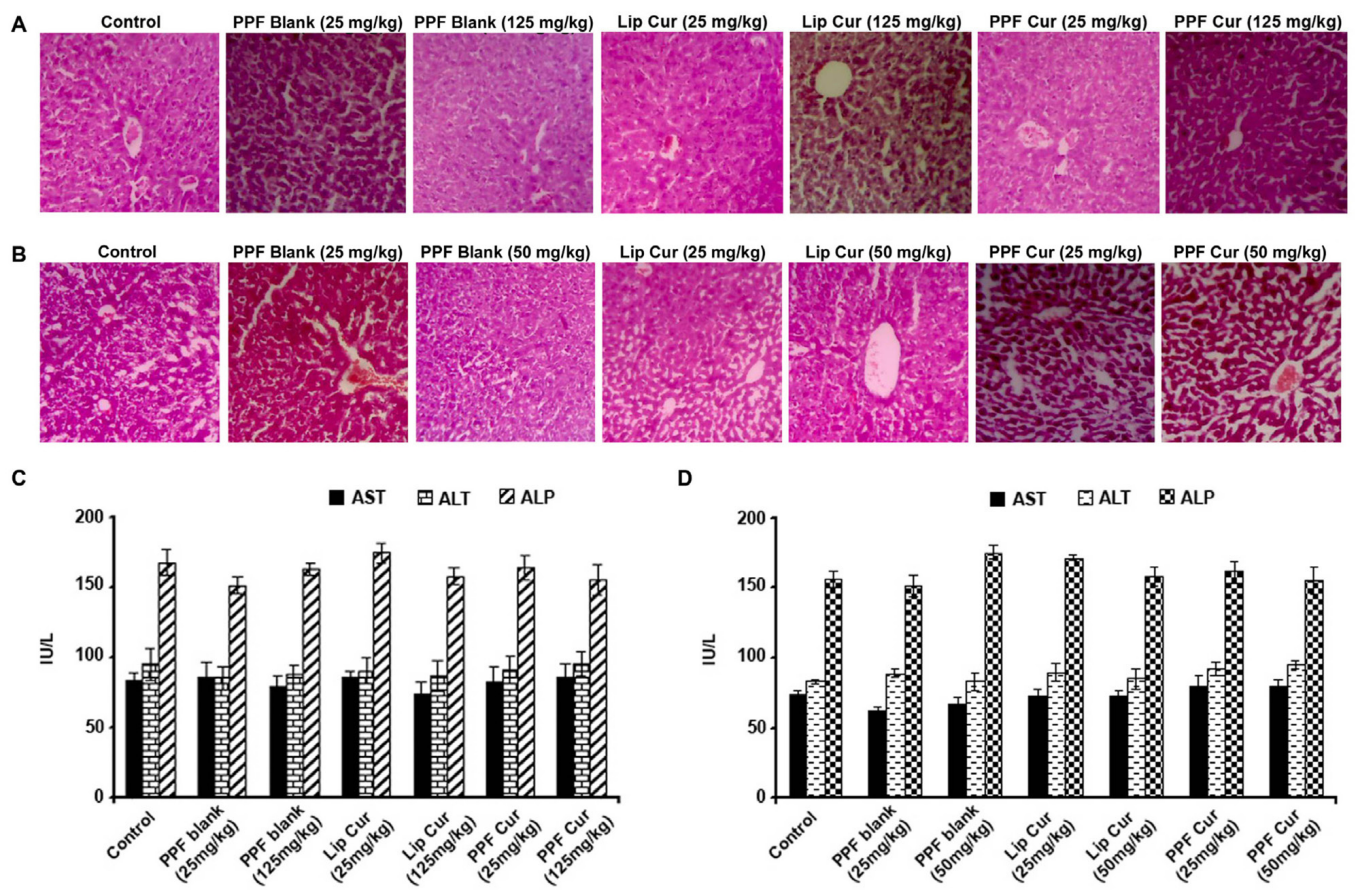
PPF curcumin is pharmacologically safe as assessed by acute and chronic toxicity studies in *Swiss albino* mice (**A**) Histopathological analysis of liver tissues of mice administered with 25 mg/kg or 125 mg/kg liposomal curcumin, PPF-curcumin or the void carrier, during acute toxicity study (7 days). (**B**) Histopathological analysis of the liver tissues of mice subjected to chronic toxicity study for 2 months using 25 mg/kg or 50 mg/kg liposomal curcumin, PPF-curcumin or void carrier. (**C**) Liver function parameters of mice subjected to acute toxicity study using 25 mg/kg or 125 mg/kg liposomal curcumin, PPF-curcumin or the void carrier. (**D**) Liver function parameters of mice subjected to chronic toxicity study using 25 mg/kg or 50 mg/kg liposomal curcumin, PPF-curcumin or the void carrier.

Our next attempt was to validate the pharmacological safety of the formulation. We conducted both acute and chronic toxicity studies in healthy *Swiss albino* mice using both liposomal curcumin and PPF-curcumin or their blank carriers. SCID mice are immuno-compromised animals, and are usually used to grow human xenografts. As far as the toxicological evaluation of the drug is concerned, it should be given to a normal healthy animal which has all the potential to defend the toxicological parameters of the drug as the final end users of all these drugs are human beings, and hence we conducted the toxicological evaluation in healthy *Swiss albino* mice. We focused on the liver histopathological analysis and liver function parameters, since nanoparticles have been reported to cause hepatotoxicity [31]. Biochemical analysis of serum samples and histopathological evaluation of liver sections were performed in animals injected with either liposomal curcumin/PPF-curcumin or their blank carriers in both acute (7 days) and chronic toxicity studies (2 months). Histopathological verification of liver tissue sections as shown in Figure 3A and 3B revealed no significant morphological changes or pathological conditions at the dosage administered. The liver tissue sections did not show any sign of fatty or irreversible liver damage. Liver function enzymes also did not show any drastic fluctuations from their normal range indicating that either PPF-curcumin or liposome curcumin does not cause any significant hepatotoxicity as indicated in Figure 3C and 3D. A similar study was also conducted in the case of paclitaxel and its combination with liposomal curcumin or PPF-curcumin, which showed that in the combination also, there were no significant histopathological manifestations as assessed by acute and chronic toxicity studies (Supplementary Figure 3A and 3B, respectively) or wide variations in liver function parameters (Supplementary Figure 3C and 3D, respectively).

### Nanoencapsulation and folic acid conjugation enhances curcumin's chemosensitization potential towards paclitaxel causing significant reduction of tumor growth *in vivo* with reduction in NF-κB and AP-1 nuclear translocation

**Figure 4:**
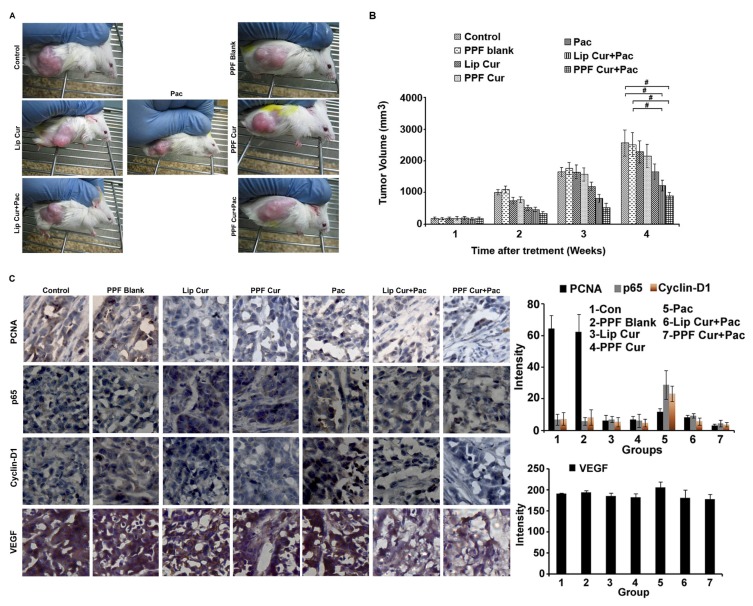
PPF-curcumin causes significant sensitization of HeLa xenograft tumors in NOD-SCID mice to paclitaxel treatment (**A**) Representative images of NOD-SCID mice bearing HeLa xenograft tumors, after 4 weeks of treatment. (**B**) Graph showing tumor volumes of NOD-SCID mice bearing HeLa xenograft tumors with or without treatment during the span of study. Combination treatment significantly (*p*-values ≤ 0.001) reduced the tumor volume in comparison to control and blank treated mice. (**C**) Immunohistochemical staining of PCNA, p65, cyclin D1 and VEGF in the tumor tissues of different treatment groups, which shows that PPF-curcumin efficiently down-regulates paclitaxel-mediated activation of survival signals in HeLa xenograft tumors in NOD-SCID mice. The quantification of the IHC images were done using ImageJ software. The results of three independent data sets are compiled as graphs. The values shown are average ± SD for 3 independent images/fields.

To assess the *in vivo* chemosensitization efficacy of PPF-curcumin towards paclitaxel, human cervical cancer xenografts of NOD-SCID mice were employed. Previous studies from our lab have shown that curcumin enhances the anti-tumor activity of paclitaxel against human cervical cancer xenograft model in NOD-SCID mice, where we used liposomal formulation [11]. Though significant tumor reduction was observed both in multistage carcinogenesis model and NOD-SCID xenograft models of cervical cancer, we could not achieve *in vivo* curcumin retention for a longer duration. The dose of paclitaxel and curcumin were used according to our earlier studies [11]. Representative images of mice from different treatment groups are shown in Figure 4A. It was interesting to note that, though curcumin or PPF-curcumin alone did not significantly reduced the tumor growth, combination of curcumin or PPF-curcumin with paclitaxel significantly suppressed tumor growth, the latter being more efficient. The tumor volume of all the treatment groups is shown in Figure 4B and indicates the enhanced chemosensitizing efficacy of PPF-curcumin in paclitaxel chemotherapy. The *p* value (*p*-value ≤ 0.001) indicates the statistical significance between control/blank and lip cur+pac/PPF+Pac combination in the 4th week. We presume that the enhanced tumor reduction in animals treated with PPF-curcumin might have been due to enhanced intra-tumoral retention of curcumin in the case of PPF-curcumin. Further, we performed the immunohistochemical analysis for the expression levels of proliferating cell nuclear antigen (PCNA), NF-κB subunit p65, pro-survival cyclin-D1 and VEGF. In animals administered with paclitaxel-PPF curcumin combination, PCNA, p65, cyclin-D1 and VEGF showed relatively low expression in tumor sections of human cervical cancer xenografts as shown in Figure 4C.

### PPF-curcumin shows enhanced tissue retention and serum concentration compared to free curcumin

**Figure 5:**
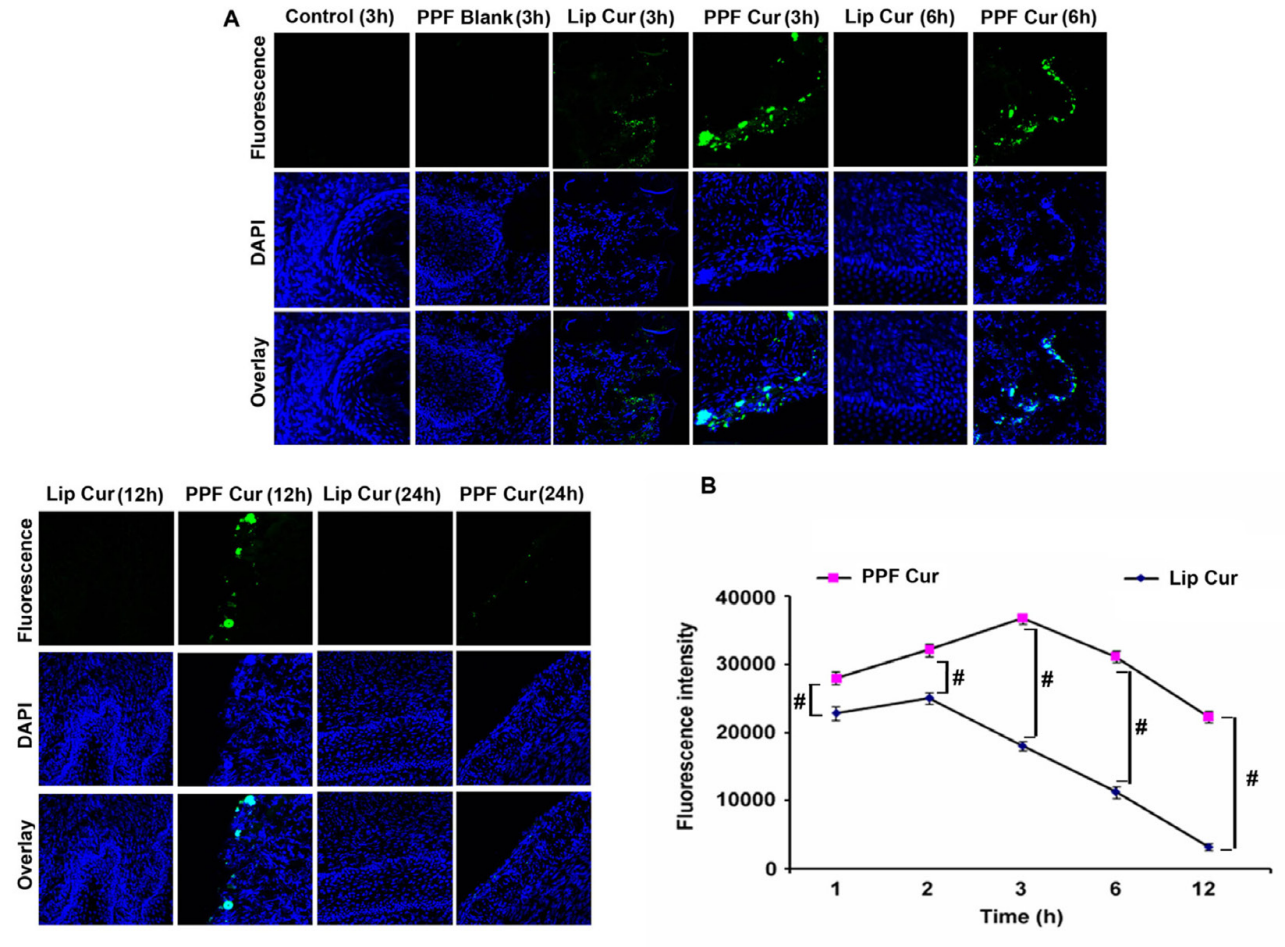
PPF-curcumin exhibits enhanced bioavailability and tissue retention of curcumin (**A**) PPF encapsulation increases the retention time of curcumin in cervical tissues. Mice were intraperitoneally injected with liposomal curcumin or PPF-curcumin (100 mg/kg) and intracellular fluorescence for curcumin in cervical tissues were assessed by confocal microscopy. Nuclei were stained with DAPI and images were captured at a magnification of 60X. (**B**) PPF-curcumin exhibits enhanced circulation of curcumin compared to liposomal curcumin. Mice were intraperitoneally injected with liposomal curcumin or PPF-curcumin (100 mg/kg). The serum was isolated and fluorescence was measured at different time intervals. ^#^indicates *p* ≤ 0.001.

As both our *in vitro* and *in vivo* results have clearly established that PPF-curcumin is superior to liposomal curcumin in chemosensitizing cervical cancer, our next attempt was to study whether folic acid conjugation has augmented the tissue retention of curcumin in cervical tissues. We compared the tissue level uptake of liposomal curcumin and PPF-curcumin in the cervix of female *Swiss albino* mice. PPF-curcumin exhibited qualitative profound fluorescence in the cervix of healthy *Swiss albino* mice from 3 h onwards and fluorescence was visible up to 24 h, while there was no detectable fluorescence in the tissue of mice treated with liposomal curcumin after 3 h as shown in Figure 5A. This might have been probably due to folic acid mediated targeting of PPF-curcumin towards FOLR1 in the cervix tissues [32]. We also recorded curcumin fluorescence in the serum of mice using spectroflourimetry to study the extent of curcumin retention *in vivo*. Serum was isolated from mice administered with liposomal curcumin or PPF-curcumin and curcumin fluorescence was recorded. Figure 5B shows that, even after 12 h there was significant curcumin fluorescence in the serum of animals injected with 100 mg/kg body weight of PPF-curcumin, whereas in the case of liposomal curcumin, there was considerable drop in the fluorescence intensity of curcumin after 6 h. *p* values (*p* ≤ 0.001) indicates the statistical significance for the same. Thus, the imaging studies and spectrofluorimetry studies clearly depict that, PPF-curcumin enables considerable uptake, improved tissue retention and prolonged circulation of curcumin *in vivo*.

### Folate receptor specific curcumin delivery using folic acid conjugated PLGA-PEG nanoparticles significantly improves the cellular uptake of curcumin, which in turn enhances its chemosensitizing potential

**Figure 6:**
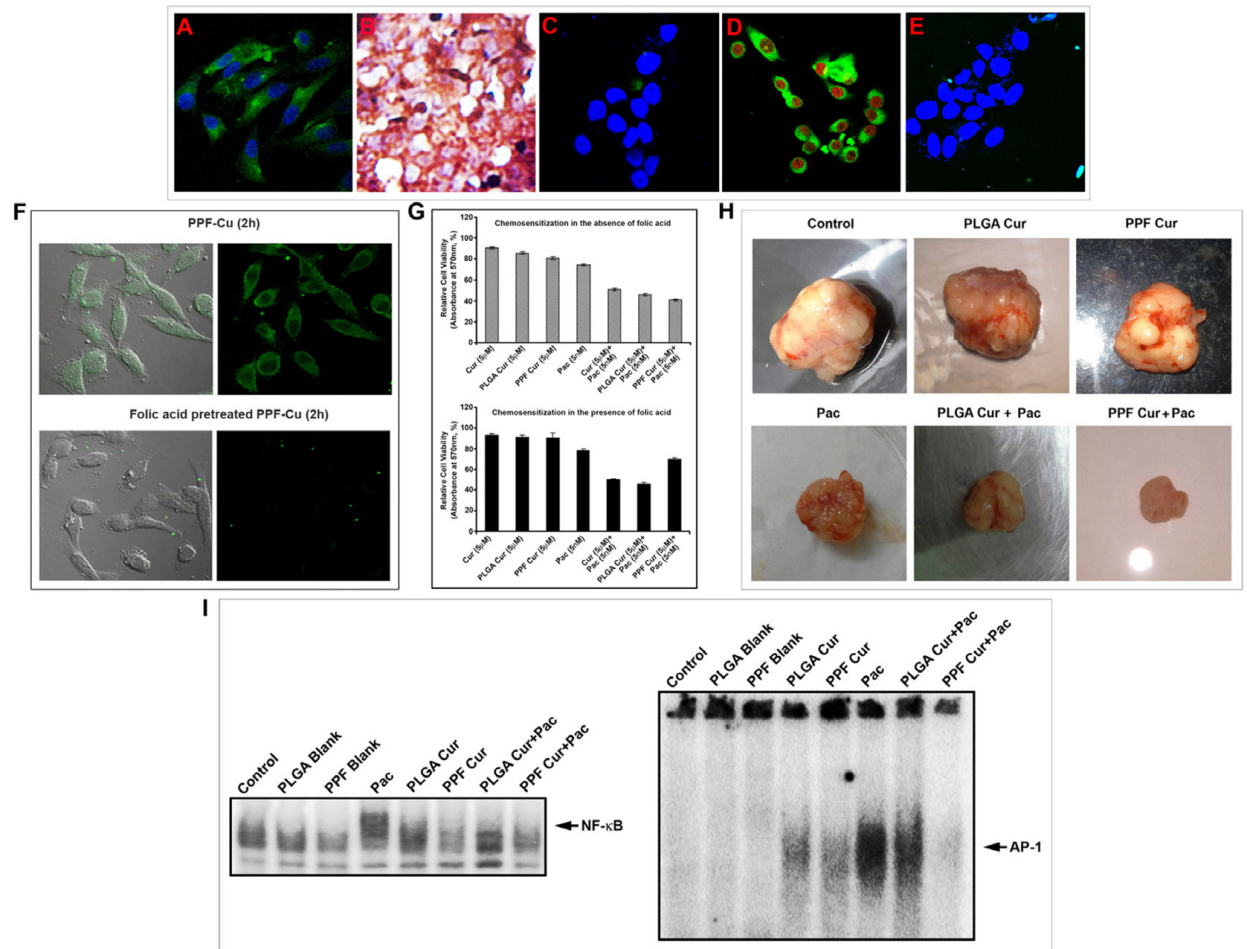
Folic acid conjugation is responsible for the enhanced chemosensitization potential of PPF-curcumin (**A**) Confocal microscopy image showing high expression of FOLR1 in HeLa cells. (**B**) Immunohistochemical staining of FOLR1 expression in HeLa xenografts. (**C**) Confocal microscopy image showing low expression of FOLR1 in HaCaT cells. (**D**) Uptake of PPF-curcumin nanoparticles in FOLR1 over-expressing HeLa cells. Cells were incubated with 5 μM PPF-curcumin nanoparticles for 2 h. Cells were fixed and nuclei were stained with PI and images were captured at a magnification of 40X. (**E**) Confocal microscopy image showing the reduced uptake of PPF-curcumin in HaCaT cells. Cells were incubated with 5 μM PPF-curcumin nanoparticles for 2 h. Cells were fixed and nuclei were stained with DAPI and images were captured at a magnification of 40X. (**F**) Uptake of PPF-curcumin in HeLa cells in the absence or presence of free folic acid. Reduced uptake of PPF-curcumin was observed in HeLa cells in the presence of free folic acid indicating FOLR1-targeted uptake of PPF-curcumin in FOLR1 over-expressing HeLa cells. Cells were incubated with free folic acid for 20 min under culture conditions as described in Materials and Methods. Later, the medium was removed and incubated with 5 μM PPF-curcumin nanoparticles for 2 h. For confocal microscopy, cells were fixed and nuclei were stained with DAPI and images were captured at a magnification of 40X. (**G**) Chemosensitization studies in HeLa cells using curcumin, PLGA-curcumin or PPF-curcumin in the presence or absence of folic acid. For cytotoxicity study, cells were cultured in the presence of 100 μg/ml and treated with curcumin or PLGA-curcumin or PPF-curcumin (5 μM) either alone in combination with paclitaxel (5 nM). Later, after 72 h of incubation, the cell viability was assessed using MTT. **(H)** Representative images of tumors from NOD-SCID mice bearing HeLa xenograft tumors with or without treatment after 4 weeks. (**I**) PPF-curcumin induced significant inhibition of NF-κB and AP-1 activation *in vivo* as assessed by EMSA.

Our next endeavor was to investigate whether the enhanced chemosensitizing efficacy of PPF-curcumin can be correlated to the over-expression of FOLR1 in HeLa cells. As already reported [19], HeLa cells exhibited high expression of FOLR1 as indicated in Figure 6A. The HeLa xenografts also exhibited very strong expression of FOLR1 as shown in Figure 6B. In contrast, the non-tumorigenic immortalized HaCaT cells did not display significant expression of FOLR1 as represented in Figure 6C. So we presumed that, the difference in cytotoxicity induced by PPF-curcumin in HeLa and HaCaT may be due to the differential expression of FOLR1 causing significantly enhanced FOLR1-mediated endocytosis and cytosolic retention of curcumin in HeLa cells in a receptor targeted fashion. Attesting our assumption, HeLa cells revealed considerable internalization of curcumin when treated with PPF-curcumin as shown in Figure 6D. Our hypothesis was further confirmed, when we could not find a significant internalization of curcumin in HaCaT cells (Figure 6E), which had minimal FOLR1 expression (Figure 6C). To confirm this inference, we pre-treated HeLa cells with folic acid before treating with PPF-curcumin and compared the fluorescence of curcumin within the cells. Figure 6F indicates that pre-treatment of the cells with free folic acid drastically reduced the uptake of PPF-curcumin in HeLa cells, which have a very high expression of FOLR1. Further, we also compared the efficacy of PPF-curcumin in chemosensitizing HeLa cells towards paclitaxel, in the presence and absence of free folic acid. Interestingly, the synergism was completely lost in the presence of free folic acid as shown in Figure 6G. However, as can be seen from the Figure 6G, free folic acid did not significantly enhance the cell viability in PPF-curcumin/paclitaxel combination. This may be because even though free folic acid quench the folate receptors, paclitaxel can still exhibit its cytotoxic activity. Moreover, the quenching of folate receptors by free folic acid is transient and hence PPF-curcumin can exert its chemosensitizing activity thereafter. To substantiate these results *in vivo*, we compared the chemosensitizing efficacy of PPF-curcumin with that of PLGA-curcumin, which lack folic acid conjugation, using HeLa xenograft model in NOD-SCID mice. Though both the formulations exhibited chemosensitizing potential, that brought about by PPF-curcumin was significantly better as indicated by Figure 6H. We compared the NF-κB and AP-1 status in various groups as our previous study had demonstrated key regulatory roles for these molecules in modulating the synergism of curcumin and paclitaxel [10]. Interestingly, PPF-curcumin induced a drastic inhibition in paclitaxel-induced DNA binding of NF-κB and AP-1 in the tumor tissues as shown by Figure 6I. The band intensities were quantified and represented in Supplementary Table 2. PLGA-curcumin also inhibited both the molecules, though to a lesser extent, demonstrating that folic acid conjugation to PLGA-curcumin nanoparticles significantly improves its chemosensitizing potential.

## DISCUSSION

**Figure 7:**
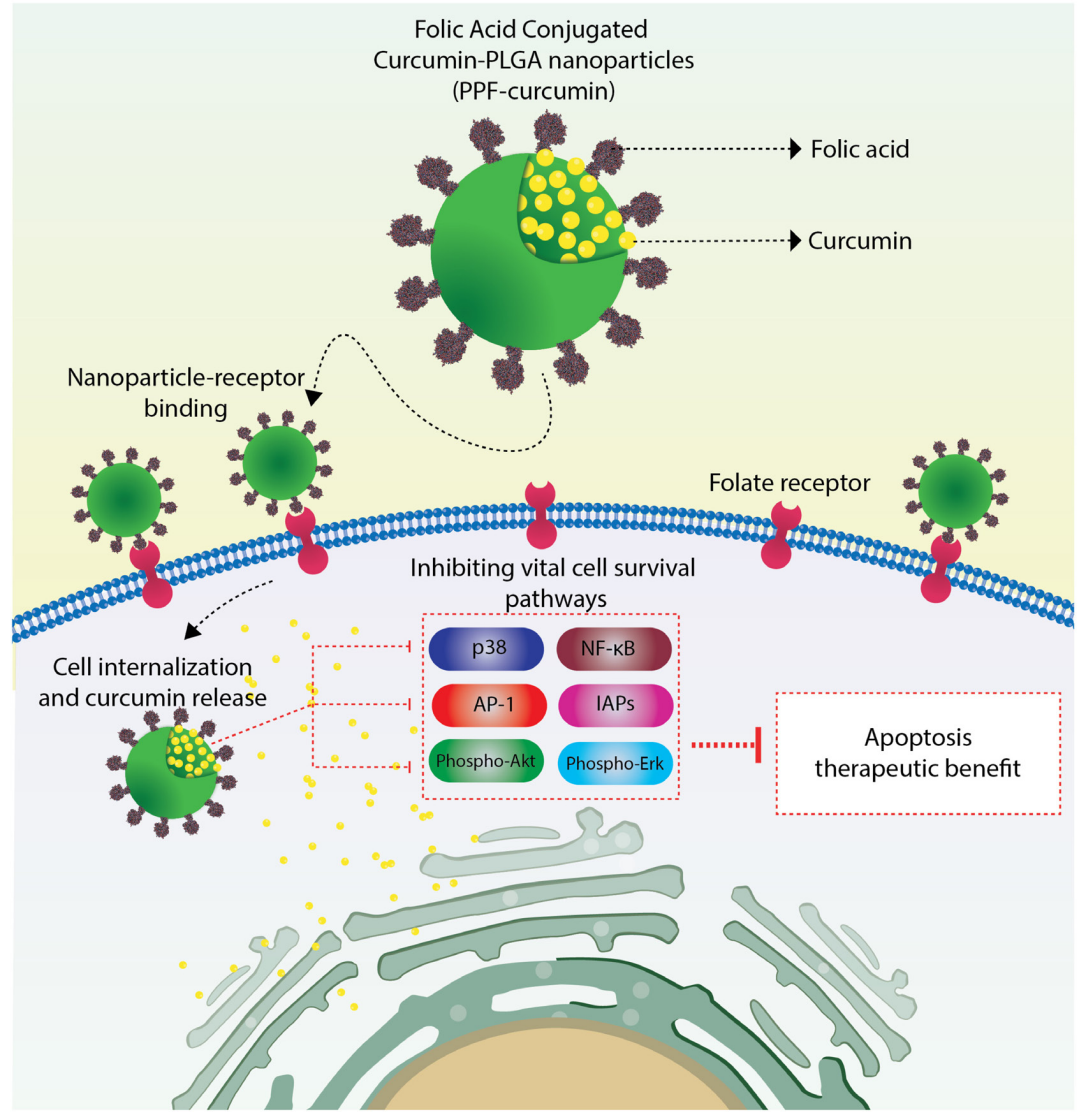
Pictorial representation of the mechanism of action of PPF-curcumin PPF-curcumin is internalized into the cancer cells via FOLR1-mediated endocytosis. In the cytosol, curcumin is released due to the degradation of PLGA nanoparticles aided by the acidic microenvironment, which further down-regulates various survival signals (p-38, NF-κB, AP-1, IAPs, phospho-Akt, phospho-Erk up-regulated by paclitaxel ultimately causing the cells to undergo apoptotic cell death.

Though paclitaxel is a widely used anti-cancer agent [33], the severity of side-effects of this drug and the solvent used for its parenteral administration are major factors that hamper its therapeutic outcome [34]. Prolonged administration of chemodrugs including paclitaxel has been shown to switch on several survival signals such as NF-κB, Akt, MAPK and AP-1, ultimately leading to chemoresistance [35]. Curcumin has been established as a successful chemosensitizer capable of down-regulating the expression of proliferation, anti-apoptotic and metastatic gene products ultimately resulting in the reversal of chemoresistance [36–38]. Both *in vitro* and *in vivo* studies conducted earlier in our laboratory have demonstrated the efficacy of curcumin in sensitizing cervical cancer cells towards paclitaxel, wherein significant down-regulation of several survival pathways was observed, though NF-κB was the key regulatory molecule [10, 11]. For both the *in vivo* tumor models, we had administered curcumin in a liposomal formulation [11]. The major drawbacks of this formulation were poor bioavailability and low tissue retention of curcumin. To overcome these limitations, we synthesized various formulations of curcumin and studied their physico-chemical characteristics and therapeutic efficacy, *in vitro* [17–19, 39]. Although none of these formulations exhibited drastic improvement in the anti-cancer potential than free curcumin, it should be mentioned that all these formulations were administered to the *in vitro* systems in aqueous dispersible suspensions, while free curcumin was administered in DMSO, which is an undesirable vehicle for *in vivo* applications. Thus, we could overcome one of the major hurdles pertaining to the hydrophobic nature of curcumin, which significantly hamper the intracellular delivery of the compound. Our next attempt was to prepare a formulation, which will help us to deliver curcumin in a target-specific manner. We selected PLGA nanoparticles as vehicle to deliver curcumin for our *in vivo* study, since PLGA is a widely accepted non-toxic material approved by FDA for human applications [17, 19]. Moreover the biodegradable nature of PLGA makes it attractive for *in vivo* applications [40–42]. Since FOLR1 is widely over-expressed in almost all cancer cells, we selected folic acid as our tumor targeting ligand. Several groups have reported the conjugation of nanoparticles with folic acid for tumor targeted nanoparticle delivery [43–45]. We conjugated folic acid to curcumin-encapsulated PLGA-PEG copolymer nanoparticles (PPF-curcumin) and compared its efficacy as a chemosensitizer for paclitaxel chemotherapy of cervical cancer, with that of liposomal curcumin [11]. Since it has been reported that, liposome formulations are internalized in cancer cells in a non-targeted fashion, which can cause curcumin accumulation in undesirable sites [46, 47], we performed a detailed investigation to evaluate and compare the chemosensitization potential of both the formulations using both *in vitro* and *in vivo* experiments. The enhanced chemosensitization potential of PPF-curcumin was evident when it induced significant enhancement in paclitaxel- mediated apoptosis and inhibition of clonogenic potential of HeLa cells compared to free curcumin. It was also interesting to see that the formulation did not chemosensitize the non-tumorigenic immortalized HaCaT cells towards paclitaxel. Moreover, the *in vivo* acute and chronic toxicity studies, confirmed that PPF-curcumin is pharmacologically safe either alone or in combination with paclitaxel. At the same time, the formulation brought about considerable augmentation in the efficacy of curcumin in down-regulating the pivotal molecules regulating the synergistic toxicity of paclitaxel and curcumin towards cervical cancer cells [10]. In conjunction with the *in vitro* studies, PPF-curcumin showed enhanced chemosensitization potential towards paclitaxel in cervical cancer xenograft models of NOD-SCID mice, than liposomal curcumin, causing significant tumor reduction *in vivo*. The tumor reduction was supported by the results of various molecular assays and immunohistochemical analyses, which indicate that PPF-curcumin in combination with paclitaxel is exceedingly successful in down-regulating the key survival pathways responsible for the uncontrolled proliferation, apoptosis evasion and tumor progression. Thus, the success of PPF-curcumin over liposomal curcumin in down-regulating the survival signals induced by paclitaxel can be attributed to its FOLR1 targeting capability, which was successfully substantiated by the drastic difference in FOLR1 expression and curcumin uptake in both HeLa and HaCaT cells, where the latter possess minimal FOLR1. This observation is further authenticated when; uptake of PPF-curcumin in FOLR1 over-expressing HeLa cells was drastically reduced by free folic acid pre-treatment. The low expression of FOLR1 and hence the low uptake of PPF-curcumin in HaCaT cells may be the reason behind the absence of synergistic cytotoxicity of paclitaxel-curcumin combination, even after delivering curcumin as PPF-curcumin. The results of both *in vitro* cytotoxicity studies and xenograft tumor reduction studies, which compared the chemosensitizing efficacy of PLGA-curcumin and PPF-curcumin towards paclitaxel chemotherapy of cervical cancer clearly demonstrated and confirmed that folic acid conjugation is the modification responsible for the extra efficacy of PPF-curcumin. Our previous study has shown that, Akt is upstream and MAPKs are downstream of NF-κB, which act as a key regulator of curcumin-mediated chemosensitizion of paclitaxel chemotherapy of cervical cancer [10]. AP-1 is a transcription factor, which is stimulated by a complex network of MAPKs of ERK, p-38 and JNK families [11]. Corroborating this fact, PPF-curcumin caused significantly better abrogation of NF-κB and AP-1, compared to PLGA-curcumin, which may be correlated with the extent of tumor reduction observed in the animals treated with PPF-curcumin. Thus in the current study, we have clearly demonstrated how folic acid conjugation to curcumin-encapsulated PLGA-PEG nanoparticles significantly improves the bioavailability and tissue retention of curcumin, thereby enhancing the efficacy of curcumin as a chemosensitizer in FOLR1 over-expressing cervical cancer cells. The proposed mechanism of action by which PPF-curcumin causes the suppression of survival signals, which leads to the chemosensitization of cervical cancer cells towards paclitaxel, is illustrated in Figure 7. PPF-curcumin is internalized into the cancer cells via FOLR1-mediated endocytosis. In the cytosol, curcumin is released due to the degradation of PLGA nanoparticles aided by the acidic microenvironment, which further down-regulates various survival signals up-regulated by paclitaxel ultimately causing the cells to undergo apoptotic cell death.

The current study gains immense significance in the scenario of accumulating evidences of chemoresistance induced by prolonged exposure of cancer cells to chemotherapeutics. Therefore, with regard to future clinical applications, the present strategy may offer promising therapeutic benefits that may be achieved at low concentrations of paclitaxel, which in turn may overcome chemoresistance and toxic side-effects, the major hurdles impeding the clinical outcome of several anti-cancer drugs.

## MATERIALS AND METHODS

### Cell lines

HeLa and HaCaT cells were procured from National Centre for Cell Sciences (NCCS), Pune, India.

### Chemicals

Curcumin, antibody against β-actin, and the horseradish peroxidase (HRP) conjugated secondary antibodies were obtained from Sigma-Aldrich (St. Louis, USA). Curcumin with a purity of ≥ 65% (HPLC) was used for our studies. Paclitaxel was obtained from Calbiochem (San Diego, CA, USA). MTT was purchased from Calbiochem (La Jolla, USA). DAPI (4′,6-Diamidino-2-phenylindole dihydrochloride), antibodies against Cox-2, PARP and Cyclin-D1 were purchased from Santacruz Biotechnology (Santa Cruz, CA, USA). The radio labelled [γ-^32^P] ATP was obtained from Bhabha Atomic Research Centre (BARC), India. The oligonucleotide probes used for electrophoretic mobility shift assays were custom synthesized (Genosys, Sigma). Dulbecco's Modified Eagle Medium (DMEM) and streptomycin sulphate were purchased from Invitrogen Corporation (Grand Island, USA). Antibodies against p-Akt, p-p42/44, p-JNK, p-P38 and monoclonal antibody against caspase-3 and rabbit polyclonal antibody against caspase-9 were purchased from Cell Signaling Technologies (Beverly, MA, USA). Immobilon Western Chemiluminescent HRP Substrate was purchased from Millipore (Billerica, MA, USA). Super Sensitive™ Polymer-HRP Detection System kit was procured from Biogenex (Fremont, CA, USA). Antibody against folate receptor (FOLR1 antibody) was purchased from Invitrogen Corporation (Grand Island, USA). All other reagents were procured from Sigma-Aldrich, unless otherwise mentioned.

### Synthesis and characterization of PLGA-curcumin and PPF-curcumin

Synthesis of PLGA-curcumin and PPF-curcumin was performed as reported earlier [19]. Poly lactic-co-glycolic acid (PLGA) 50 : 50 (lactic/glycolic ratio) having MW 24 000 Da to 38 000 Da, poly ethylene glycol (PEG) bisamine MW 3000 Da, N-hydroxy succinimide (NHS) MW 115.09, dicyclohexylcarbodiimide (DCC) MW 206.3, pyridine, folic acid, dichloro methane (DCM), dimethyl sulfoxide (DMSO), acetone, curcumin, acridine orange and ethidium bromide were procured from Sigma (Steinheim, Germany). Polyvinyl alcohol (PVA) 87% hydrolysed was purchased from SD fine (Mumbai, India). For morphological analysis of PPF-curcumin using transmission electron microscope (TEM, JEOL 1011, Japan), nanoparticle suspension was diluted in Milli-Q^®^ (Millipore Corporation, Billerica, MA) water at 25°C and drop casted onto formvar coated grids.

### Drug treatment

Cells were pretreated with curcumin or PPF-curcumin (5 μM) either alone or in combination with paclitaxel (5 nM), 2 hours prior to paclitaxel (5 nM) treatment. The DMSO concentration in all the experiments was maintained <0.25%.

### MTT assay

Cell viability after drug treatment was determined by MTT assay. Cells (~2.5 × 10^3^) seeded in 96-well plate were treated with drugs either alone or in combination and MTT assay was performed as described earlier [11], after 72 hours of incubation, post paclitaxel treatment. Untreated cells, PPF and PLGA blank treated cells were used as controls.

### Clonogenic assay

To compare the anti-clonogenic potential of different formulations of curcumin either alone or in combination with paclitaxel clonogenic assay was performed as reported earlier [19].

### Western blot analysis

The whole cell lysate was prepared from the cells treated with or without drug and subjected to Western blot analysis as reported earlier [19].

### Chromatin condensation assay

Chromatin condensation assay was performed in drug treated cells after 24 hours of post drug treatment followed by fixing the cells and staining the nucleus using DAPI. The stained cells were mounted on a glass slide using Flouromount^TM^ aqueous mounting medium (Sigma Aldrich, USA) and images were taken using a fluorescent microscope.

### Cellular uptake of PPF-curcumin in HeLa cells

Briefly, 2 × 10^4^ cells were grown on cover slips placed in 24 well plates. Once the cells attained 80% confluency, they were treated with 5 μM PPF-curcumin for 2 h. The cells were washed with PBS and fixed using 4% paraformaldehyde. The nuclei were stained using propidium iodide for 5 min and were mounted using DPX. Cells were then examined for intracellular fluorescence of curcumin using confocal laser scanning microscope in the FITC channel (488 nm) and images were captured at 40X.

### Cellular uptake of PPF-curcumin in HaCaT cells

Briefly, 2 × 10^4^ cells were grown on cover slips placed in 24 well plates and treated with 5 μM PPF-curcumin for 2 h. The cells were washed with PBS and fixed using 4% paraformaldehyde. The nuclei were stained using 1 μg/ml DAPI for 10 min and were mounted using DPX. Cells were then examined for intracellular fluorescence of curcumin using confocal laser scanning microscope in the FITC channel (488 nm) and images were captured at 40X.

### FOLR1 quenching studies using confocal microscopy

Approximately, 2 × 10^4^ cells were grown on cover slips placed in 24 well plates. Later, they were incubated with PPF-curcumin with or without folic acid pre-treatment (100 μl of 4 mg/ml folic acid for 20 min), which has earlier proved to quench FOLR1 [48–50]. Later, the medium was removed, rinsed with PBS, fixed using 4% paraformaldehyde, stained with DAPI and observed at 40X under confocal microscope using 488 nm laser to record curcumin fluorescence.

### Cell viability of HeLa cells with and without folic acid pre-treatment

Approximately, ~2.5 × 10^3^ cells were seeded in 96-well plate and treated with curcumin or PLGA-curcumin or PPF-curcumin (5 μM) either alone in combination with paclitaxel (5 nM) in the presence or absence of free folic acid (100 μg/ml). Later, after 72 h of incubation, the cell viability was assessed using MTT cell viability assay as described earlier.

### Confocal microscopy for FOLR1 staining

HeLa or HaCaT cells were seeded on cover-slips at a density of 5000 cells per well in a 24 well cell culture plate. Later, the cells were fixed using 4% paraformaldehyde and incubated with rabbit polyclonal antibody against FOLR1 at 4–8°C, followed by FITC conjugated secondary antibody. Later, the cells were washed and stained with DAPI and observed at 40X under confocal microscope using 488 nm laser.

### *In vivo* studies

The methods were carried out in accordance with the Committee for the Purpose of Control and Supervision of Experiments on Animals guidelines and all experimental protocols were approved by Institutional Animal Ethical Committee of Rajiv Gandhi Centre for Biotechnology.

### Toxicity studies

Acute (7 days) and chronic (60 days) toxicity studies were carried out in female *Swiss albino* mice, 6 weeks of age. For acute toxicity study, liposome curcumin, PPF-curcumin (25 mg/kg and 125 mg/kg, 5 mice per group) either alone or in combination with paclitaxel in cremophor (10 mg/kg, 5 mice per group) or their corresponding void carriers were administered through intraperitonial injection. The mice were observed continuously for 1 h for any gross behavioral changes and death, and intermittently for the next 6 h and 24 h after dosing over a period of 7 days. For chronic toxicity study, liposome curcumin or PPF-curcumin (25 mg/kg and 50 mg/kg, 5 mice per group) either alone or in combination with paclitaxel in cremophor (10 mg/kg, 5 mice per group) or their corresponding void carriers were administered through intra-peritoneal injection (IP) every alternate day over a period of 14 days followed by a twice weekly regimen for 2 months. All mice were euthanized using CO_2_ at the end of the experiment, and blood was collected by cardiac puncture. Serum was isolated by centrifuging the coagulated blood samples at 1500 rpm for 10 min. Liver function parameters (AST/SGOT, ALT/SGPT) were recorded to assess the hepatotoxicity. The biochemical assays were conducted at Iype's Diagnostics, Thiruvananthapuram.

### *In vivo* bioavailability studies

Female *Swiss albino* mice (6 weeks of age) were intraperitoneally injected with either liposomal curcumin or PPF-curcumin (100 mg/kg) or their void polymer carriers. The animals were sacrificed using CO_2_ after the desired time interval and blood was collected by cardiac puncture. Cervical tissues were collected, washed in PBS and were immediately cryosectioned without fixation. The tissue sections were stained using DAPI and mounted using Flouromount^TM^. The images were recorded using Nikon A1R confocal microscope at FITC channel (488 nm) and images were analyzed with NIS elements software.

### Tumor reduction studies using NOD-SCID mice bearing xenograft

Tumor reduction studies were conducted as reported earlier [11]. Female NOD-SCID (NOD.CB17-Prkdc*^scid/J^*) mice of age 6–8 weeks were used for the experiment. Tumors were induced by subcutaneous injection of HeLa cells (~5 × 10^6^ cells in 100 μl 1X PBS) in the lower right flank of mice and were allowed to grow for a period of time (approximately 15 days) to a size of approximately 100–150 mm^3^ as measured by vernier calipers. The animals were then randomly grouped into 9 groups of 5 each: Control, PLGA blank, PPF blank, liposomal curcumin, PPF-curcumin, paclitaxel, liposomal curcumin + paclitaxel, PLGA-curcumin+ paclitaxel and PPF-curcumin + paclitaxel group. Liposomal curcumin, PLGA-curcumin or PPF-curcumin (equivalent to 25 mg/kg curcumin, on alternate days) and paclitaxel (10 mg/kg doses twice weekly) were injected intraperitonially. Tumor volume was measured once in a week. Animals were sacrificed after 40–45 days and tumor samples were collected for further analyses.

### Histopathology and immunohistochemistry

The mice tissues were collected and cryosectioned. Immunohistochemical analysis of various proteins in the xenograft tumor tissue sections was performed using the detection kit, as per manufacturer's protocol Super Sensitive Polymer-HRP IHC Detection System (Biogenex, CA, USA). The images were taken using Leica DM 1000 microscope. The results were interpreted by Dr. Sankar Sundaram, Deparatment of Pathology, Government Medical College Hospital, Kottayam.

### Electrophoretic mobility shift analysis

Nuclear extracts from tissue samples or cultured cells were prepared and EMSA was performed to evaluate DNA-binding activity of NF-kB or AP-1 as described in already reported studies [11].

### Statistical analysis

Data represent three independent sets of experiments. The error bars represent ±S.D. Statistical analysis was undertaken using Prism 5.0c Software. A two-tailed *t*-test or a one-way ANOVA was performed when comparing two groups or more than two groups, respectively. Statistical significance was defined as *P* < 0.05. Data are shown as mean ± SD. Two-way ANOVA followed by Tukey's post hoc *t-*test analysis was used for statistical comparison between different groups. ^#^means *P* ≤ 0.001 ^*^means *P* ≤ 0.01.

## SUPPLEMENTARY MATERIALS FIGURES AND TABLES


