# Supplementary material for: Folic acid conjugation improves the bioavailability and chemosensitizing efficacy of curcumin-encapsulated PLGA-PEG nanoparticles towards paclitaxel chemotherapy
Source: Oncotarget. 2017 Nov 10;8(64):107374–89. doi: 10.18632/oncotarget.22376 (PMC5746074; doi:10.18632/oncotarget.22376)
Supplement: Supplementary file 1 [file oncotarget-08-107374-s001.pdf]

# Folic acid conjugation improves the bioavailability and chemosensitizing efficacy of curcumin-encapsulated PLGA-PEG nanoparticles towards paclitaxel chemotherapy

## SUPPLEMENTARY MATERIALS

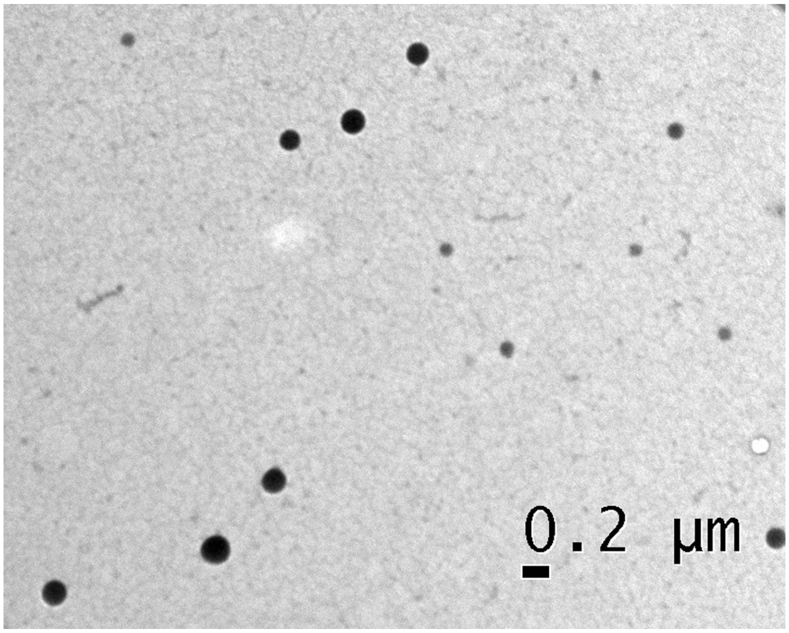


**Supplementary Figure 1: TEM image of PPF-curcumin.** The samples of the nanoparticle suspension in Milli-Q® (Millipore Corporation, Billerica, MA) water at 25°C were dropped onto formvar coated grids, and were allowed to dry completely. Images were taken and the size distributions of the various nanoparticles were analyzed using a particle size analyzer (Beckman Coulter Delsa Nano Particle Analyzer).

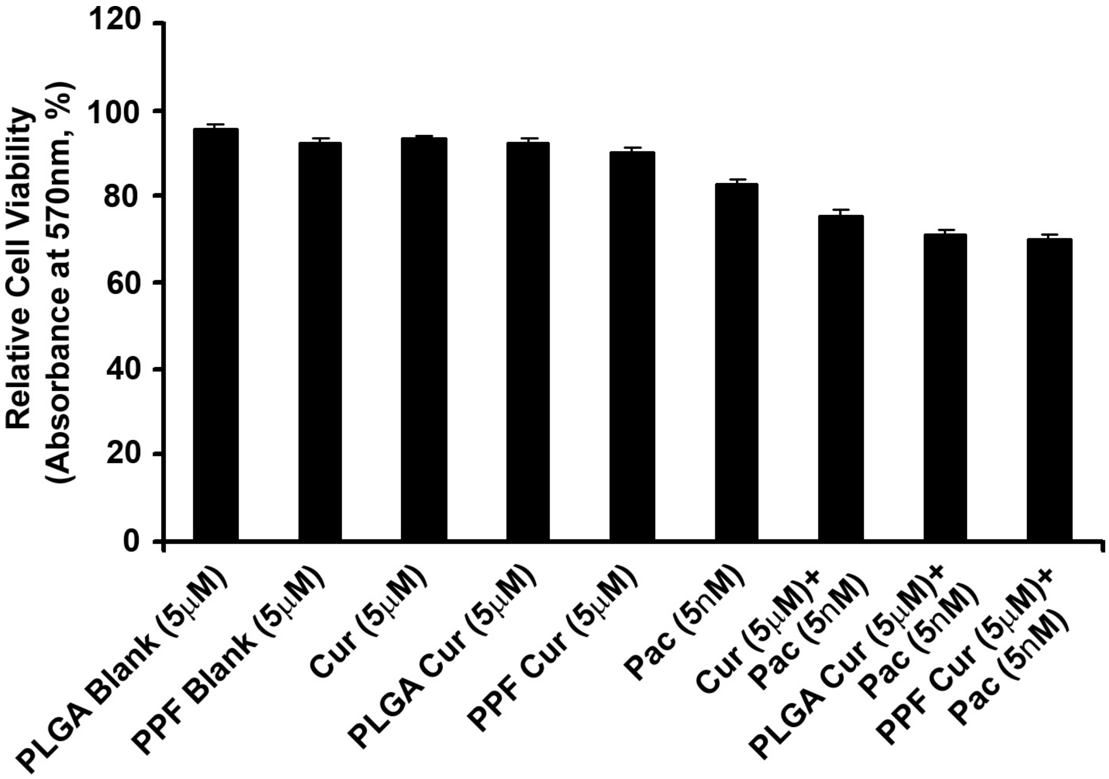


**Supplementary Figure 2: PPF-curcumin did not sensitize HaCaT cells towards paclitaxel.** The cells were treated with free curcumin/PLGA-blank/PPF-curcumin either alone or in combination with paclitaxel for 72 h after pre-treating with curcumin/PPF-curcumin and cell viability assay was performed using MTT.

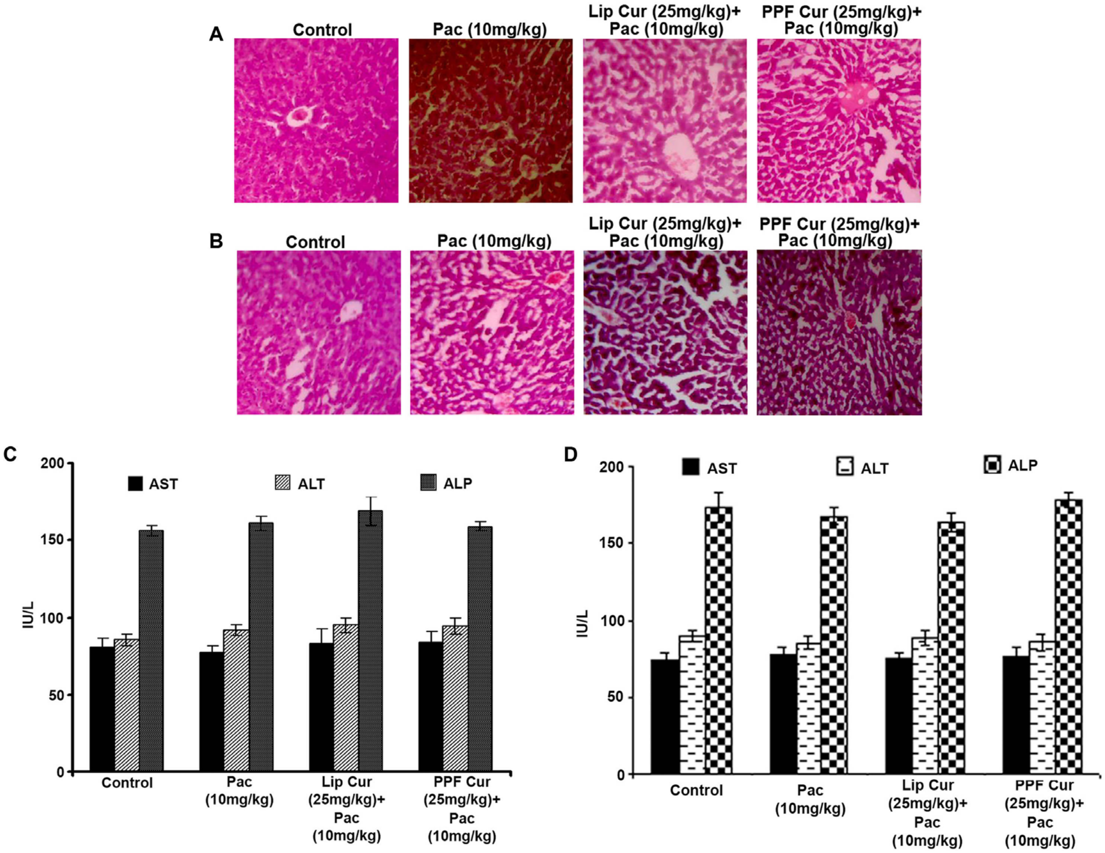


**Supplementary Figure 3: Liposome curcumin or PPF-curcumin does not induce acute or chronic toxicity in combination with paclitaxel.** (**A**) Histopathological analysis of liver tissues of mice subjected to acute toxicity for 7 days study using paclitaxel 10 mg/kg, or its combination with liposomal curcumin or PPF-curcumin (25 mg/kg). (**B**) Histopathological analysis of liver tissues of mice subjected to chronic toxicity for 2 months using paclitaxel 10 mg/kg or its combination with liposomal curcumin or PPF-curcumin (25 mg/kg). (**C**) Liver function parameters of mice subjected to acute toxicity study using paclitaxel 10 mg/kg or its combination with liposomal curcumin or PPF-curcumin (25 mg/kg). (**D**) Liver function parameters of mice subjected to chronic toxicity study using paclitaxel 10 mg/kg or its combination with liposomal curcumin or PPF-curcumin (25 mg/kg).

**Supplementary Table 1: Combination index of curcumin/PPF curcumin and paclitaxel on HeLa cells**

| Combinations | | Fa | CI | Effect | % viability |
|---|---|---|---|---|---|
| Cur (μM) | Pac (nM) | | | | |
| 1 | 1 | 0.183 | 0.727 | Synergistic | 81.7 ± 1.46 |
| 5 | 5 | 0.472 | 0.685 | Synergistic | 52.8 ± 2.61 |
| 10 | 10 | 0.639 | 0.601 | Synergistic | 36.1 ± 1.54 |
| PPF Cur (μM) | | | | | |
| 1 | 1 | 0.209 | 0.447 | Synergistic | 79.1 ± 1.80 |
| 5 | 5 | 0.573 | 0.315 | Synergistic | 42.7 ± 2.20 |
| 10 | 10 | 0.656 | 0.412 | Synergistic | 34.4 ± 3.61 |

The table indicates the combination index values and the effect of the various combinations on HeLa cells. HeLa cells were subjected to MTT assay using the different combinations mentioned and the cell viability was calculated as described in the Materials and Methods. The combination index was calculated using the method proposed by Chou and Talalay [51]. CI <1 is indicative of Synergistic combination; C>1 indicates Antagonistic effect and CI =1 indicates an additive effect. Abbreviations: Cur=Curcumin; PPF Cur=PPF-curcumin; Pac=Paclitaxel; Fa=Fraction affected; CI= Combination Index.

**Supplementary Table 2: EMSA band density in tissue nuclear extracts**

| Band density | PLGA-blank | PPF-blank | PLGA-Cu | PPF-Cu | Pac | PLGA-Cu+Pac | PPF-Cu+Pac |
|---|---|---|---|---|---|---|---|
| NF- κB | 0.8 | 0.75 | 1.3 | 1 | 1.9 | 1.13 | 0.8 |
| AP-1 | 1 | 1 | 1.8 | 2.0 | 3.9 | 3.1 | 1.5 |

The table indicates the fold change of the bands compared with the control (first band) for EMSA for NF- κB and AP-1 in the tissue nuclear extracts.
